# Segmenting Clinicians’ Usage Patterns of a Digital Health Tool in Resource-Limited Settings: Clickstream Data Analysis and Survey Study

**DOI:** 10.2196/30320

**Published:** 2022-05-09

**Authors:** Kate Miller, Julie Rosenberg, Olivia Pickard, Rebecca Hawrusik, Ami Karlage, Rebecca Weintraub

**Affiliations:** 1 Better Evidence Ariadne Labs Boston, MA United States; 2 Department of Health Policy and Management Harvard TH Chan School of Public Health Boston, MA United States; 3 Division of Global Health Equity Brigham and Women's Hospital Boston, MA United States

**Keywords:** informatics, clinical decision support tools, low-income settings, provider behavior, digital health, behavioral segments, clinicians, clickstream data, web usage mining

## Abstract

**Background:**

Evidence-based digital health tools allow clinicians to keep up with the expanding medical literature and provide safer and more accurate care. Understanding users’ online behavior in low-resource settings can inform programs that encourage the use of such tools. Our program collaborates with digital tool providers, including UpToDate, to facilitate free subscriptions for clinicians serving in low-resource settings globally.

**Objective:**

We aimed to define segments of clinicians based on their usage patterns of UpToDate, describe the demographics of those segments, and relate the segments to self-reported professional climate measures.

**Methods:**

We collected 12 months of clickstream data (a record of users’ clicks within the tool) as well as repeated surveys. We calculated the total number of sessions, time spent online, type of activity (navigating, reading, or account management), calendar period of use, percentage of days active online, and minutes of use per active day. We defined behavioral segments based on the distributions of these statistics and related them to survey data.

**Results:**

We enrolled 1681 clinicians from 75 countries over a 9-week period. We based the following five behavioral segments on the length and intensity of use: short-term, light users (420/1681, 25%); short-term, heavy users (252/1681, 15%); long-term, heavy users (403/1681, 24%); long-term, light users (370/1681, 22%); and never-users (252/1681, 15%). Users spent a median of 5 hours using the tool over the year. On days when users logged on, they spent a median of 4.4 minutes online and an average of 71% of their time reading medical content as opposed to navigating or managing their account. Over half (773/1432, 54%) of the users actively used the tool for 48 weeks or more during the 52-week study period. The distribution of segments varied by age, with lighter and less use among those aged 35 years or older compared to that among younger users. The speciality of medicine had the heaviest use, and emergency medicine had the lightest use. Segments varied strongly by geographic region. As for professional climate, most respondents (1429/1681, 85%) reported that clinicians in their area would view the use of a online tool positively, and compared to those who reported other views, these respondents were less likely to be never-users (286/1681, 17% vs 387/1681, 23%) and more likely to be long-term users (655/1681, 39% vs 370/1681, 22%).

**Conclusions:**

We believe that these behavioral segments can help inform the implementation of digital health tools, identify users who may need assistance, tailor training and messaging for users, and support research on digital health efforts. Methods for combining clickstream data with demographic and survey data have the potential to inform global health implementation. Our forthcoming analysis will use these methods to better elucidate what drives digital health tool use.

## Introduction

Digital health tools, including evidence-based clinical resources (EBCRs), can enhance health workers’ knowledge base and skill set and can provide decision-making support. They improve diagnostic accuracy and promote quality, efficient care by allowing clinicians to integrate evidence-based information directly into clinical decision-making [1,2]. Recent research from the United States demonstrates that the use of UpToDate, a leading commercial EBCR, increased clinicians’ performance on standardized exams and reduced patients’ average length of stay and risk-adjusted mortality rates at nonteaching hospitals [3,4]. Observational research from several low-income countries shows that the use of an EBCR is associated with either improved outcomes or process measures [5-7].

However, in many resource-limited health care settings, the subscription cost of commercial EBCRs is prohibitive. Over the past decade, the Better Evidence program at Ariadne Labs has collaborated with UpToDate to distribute free subscriptions to clinicians serving vulnerable populations. The program now reaches a diverse group of over 30,000 medical professionals in more than 120 countries annually. Although most donation recipients do integrate this tool into their practices [8], usage still varies among clinicians. To maximize the impact of digital health tools, we seek to understand the barriers and facilitators that shape the way clinicians use (or do not use) EBCRs, so that we can tailor our outreach and interventions to encourage engagement with and the sustained use of these tools [9,10].

Social scientists and program implementers are aware of the effects of behavioral heterogeneity and have used psychographic-behavioral segmentation to group study subjects based on their preferences, beliefs, and self-reported behaviors [11]. We believe that online behavior, which has fueled extensive behavioral modeling in computer science [12,13], provides another useful dimension for behavioral segmentation in global health. Segmenting users based on online behavior may allow us to better understand, predict, and support the uptake of EBCRs among different populations.

Websites and apps generate clickstream data, which include each click from every user, identifying which pages users visit and when users visit them. Although the field of e-commerce extensively mines these data to understand online consumer behavior, analyses of clickstream data from digital health tools have been scarce [14-16]. To our knowledge, no studies have defined user segments based on clickstream data from EBCR users across the globe, possibly due to the difficulty of generating the data structure and analytics for these large, detailed data sets.

The goal of this research was to define behavioral user segments among EBCR users around the globe, with the larger aim of understanding that behavior and tailoring the implementation of digital health tools to encourage uptake, including identifying clinicians who may need assistance and designing training and messaging for them. We hope that this research will also provide a useful method and approach for studying other digital health efforts. To this end, we conducted a study of clinicians, who were awarded with donated UpToDate subscriptions, and collected data from the following two data sources: (1) clickstream data from the back end of UpToDate and (2) a baseline user survey on demographics, access to internet-enabled devices, and the professional climate around using EBCRs in practice. Herein, we report on our process for defining behavioral segments from the raw clickstream data and how these segments relate to demographics and baseline survey responses.

## Methods

### Data Use Agreement and Ethical Approvals

We worked with UpToDate to design a fair data use agreement. We received ethical approval from the Partners Human Research Committee and Harvard TH Chan School of Public Health institutional review boards and designed informed consent language that covered the collection of both the survey and clickstream data for research purposes (institutional review board approval: June 19, 2017, under protocol 2017P001045).

### Study Participants

Clinicians were eligible for the research study if they applied for a new UpToDate donation on the Better Evidence website [17] during the 9-week study enrollment period (March 1 to May 4, 2018), met the eligibility criteria for the donation program, and provided informed consent. The donation program eligibility criteria included being a physician, surgeon, or physician's assistant; having at least intermittent internet access; and being able to complete the application in English. In addition, clinicians had to demonstrate a need for a donation by working or volunteering at a public or nonprofit entity, attesting that neither they nor their organization could afford UpToDate otherwise, and submitting personal statements describing the mission of their organization and the communities they serve. Using the standard application review process, staff at Better Evidence and UpToDate then vetted each application to confirm the declaration of need. Clinicians could be based in any country outside the United States as long as they worked in low-resource settings. We did not actively recruit clinicians; they typically visited the Better Evidence website after learning about the donation program from a colleague.

### Clickstream Data

After approval for an UpToDate donation, clinicians received a subscription activation link by email. We tracked their clicks on UpToDate for 12 months, following the date that their subscription activation link was sent to them. Clicks were recorded across all mobile and desktop applications as well as during offline use.

**Figure 1:**
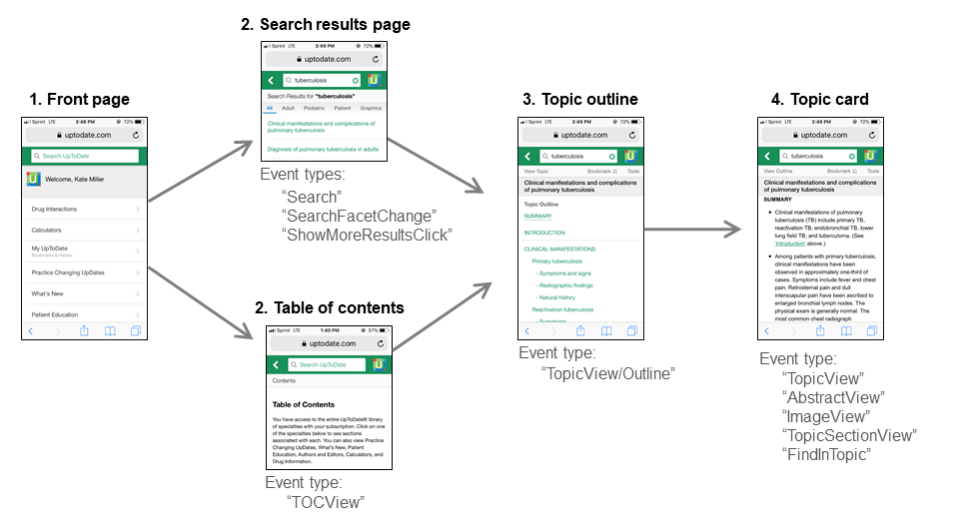
UpToDate user interface, typical navigational path, and selected click events.

Each row in the data set represented an individual click by an individual clinician. We used the following three variables per row in this analysis: an anonymized unique identifier for each clinician; the time stamp of the click (recorded to the millisecond); and the click’s “event type,” which was assigned by UpToDate. An event type defines the action taken or material presented by each click (Figure 1). For instance, if a clinician typed a term in the search window and clicked “Search,” the click was labeled with the event type “Search.” If the clinician navigated to the central table of contents, the click was labeled with the event type “TOCView.” If the clinician selected a particular topic, the click was labeled with the event type “TopicView/Outline.” If the clinician clicked on a topic card (which contained medical content), the click was labeled with the event type “TopicView.”

Other event types included making changes to search settings (“SearchFacetChange”) or viewing a specific section within a topic (“TopicSectionView”). Offline use and text-mode use also generated specific event types.

We removed all double clicks (clicks within 500 milliseconds of each other) and any cases in which a clinician had exactly 1 click in the data set.

### Defining Activities

We categorized all event types into the following three main activities: navigating, reading, and account management. Navigating events included searches and any movement toward the medical content, such as clicking through the table of contents. Reading events included any exposure to medical content in the form of topic cards, abstracts, images, or drug interactions. Additionally, we viewed events that involved printing or sharing medical content as a continuation of reading events and classified them as reading events. Account management events included changes to account information, the creation of bookmarks, and the setting of preferences. Finally, some event types, such as “ExternalLinkClick,” signaled the end of activities within UpToDate and did not need to be classified as an activity. We present the full set of 42 event types and their classifications in Multimedia Appendix 1.

### Defining User Sessions

Next, we identified clinician “sessions”—discrete groupings of clicks that represented a single, continuous interaction with UpToDate. The clickstream data did include a system-generated session variable, but these sessions often overlapped in time and were difficult to interpret, as we did not have access to the algorithm that generated them. Moreover, in the literature on session identification from clickstream data, no consensus yet exists on standard methods [18,19]. For these reasons, we defined our own simple method of grouping clicks into sessions of measurable activity.

**Figure 2:**
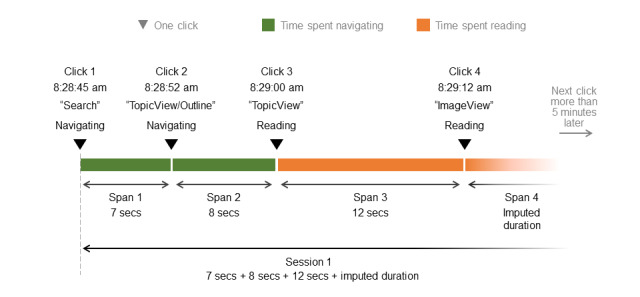
From clicks to sessions.

We first grouped together clicks with less than 5 minutes between them (Figure 2). If clicks occurred more than 5 minutes apart, we assumed that the first session had ended and that the second click was the start of a new session. Using such a time-out value is a common way to define sessions, and although longer times of up to 20 minutes are often used, we chose a shorter period because UpToDate is designed for use at the point of care when time and attention are limited [20].

In the time span between 2 clicks, we assumed that the clinician was actively engaged in the activity signaled by the first click in the pair, that is, navigating, reading, or account management. However, we could not assume that the activity ended with the final click, as that would assume that the clinician spent no time on performing the final activity, nor could we assume that the entirety of the time (sometimes days or weeks) between clicks represented the time spent on the final activity, given that the app may remain open. Thus, in the example shown in Figure 2, click 4 was the final click in the session but did not necessarily signal the end time of the session because the clinician likely spent some time actively engaged with the final reading activity.

We modeled the duration of these final activity periods as follows:

Duration = β_0_ + β_1_ Activity + Ɛ

where “Activity” was a nominal variable with 3 values (navigating, reading, or account management), and we included individual clinicians as a random effect. The duration of activity periods with a known length followed a log distribution, so we used a Box Cox power transformation for the dependent variable, as follows:

Duration_Transformed_ = (Duration^λ^ – 1)/ λ

where λ was estimated at –0.25. We back-transformed all predicted activity period durations to a natural scale. Finally, we aggregated activity periods into sessions with known start and end times and identified the time spent on navigating, reading, or account management within each span.

### Defining Segments

We assessed sessions at the level of the individual clinician, resulting in several statistics describing each clinician’s online behavior over the subscription year, as follows:

- Number of sessions: total number of sessions over the full year
- Time online: total duration of all sessions summed over the full year
- Time spent per activity: percentage of total time online spent on each activity (navigating, reading, or account management)
- Lag: number of days between clinician’s receipt of activation email and clinician’s first click
- Period of use: number of days between a clinician’s first and last clicks
- Active days: percentage of days in the period of use with at least 1 session
- Rate of use: average minutes spent online per active day during the period of use
- Lapse: number of days between a clinician’s last click and the end of one year, defined as 365 days after the activation email was sent to the clinician

By design, the sum of the lag, period of use, and lapse days equaled 365 for every clinician. We defined “dropouts” as those who stopped using UpToDate for a period of 6 weeks or more before the end of the year.

We used these statistics describing individual use to define behavioral segments. We did not follow formal statistical rules to build these segments but aimed for definitions that reflected the observed distributions and would be programmatically meaningful, be simple to explain, and be easily calculated in future data sets.

### Surveys

We created the baseline survey in REDCap (Research Electronic Data Capture; a online software platform created by Vanderbilt University). The survey explored expected barriers to and facilitators of the use of the EBCR (see Multimedia Appendix 2 for survey questions). We pilot-tested the survey with approximately 1 dozen clinicians from 4 countries for clarity, wording, response options, ability to answer, and acceptability, and integrated it into the application for a donated UpToDate subscription. We linked the survey responses and clickstream data through a unique identifier.

We presented the distribution of the segments by the following demographic traits: gender, age group, specialty, patient load per week, and geographic region of the world (see Multimedia Appendix 3 for a list of countries). We also presented the segment distributions by the baseline survey responses to questions about access to a device and professional context. For the survey responses, we adjusted the segment distributions by demographic traits by using multinomial logistic regression. We performed no statistical tests of the differences in segment distribution, both to avoid multiple testing and to recognize that our convenience sample may not be representative of all EBCR users in resource-limited settings. We performed all analyses by using SAS version 9.4 (SAS Institute).

## Results

### Measures of Individual Use

**Figure 3:**
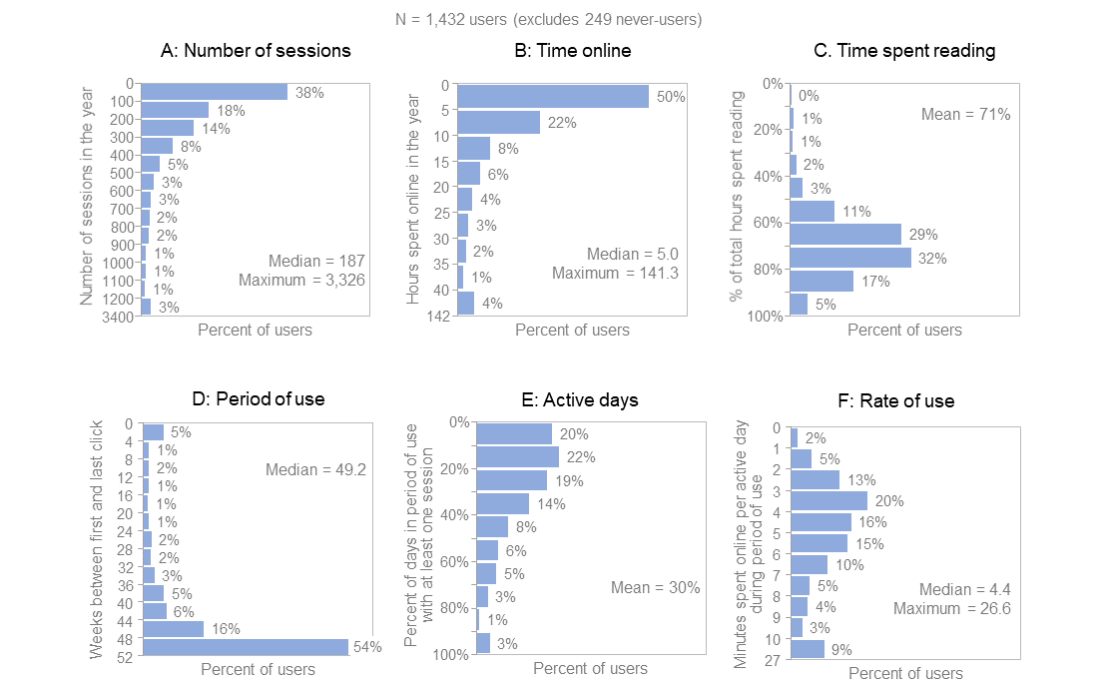
Measures of online behavior.

Of the 1681 clinicians enrolled in the study, 249 (14.8%) never used UpToDate, and the other 1432 (85.2%) did appear in the clickstream data, which included 3,059,985 clicks; these aggregated to 398,089 sessions (Figure 3). Among those who ever used UpToDate, 38% (544/1432) had 100 or fewer sessions over the year, and 18% (258/1432) had between 100 and 200 sessions (panel A). The median number of sessions was 187, and 3% (43/1432) of clinicians had more than 1200 sessions, with a maximum of 3326 sessions for 1 particular clinician. Half of the clinicians (716/1432, 50%) spent up to 5 hours total on UpToDate over the year (panel B), while the other half (716/1432, 50%) spent more time on UpToDate—up to a maximum of 141.3 hours. Clinicians spent an average of 71% of their time reading, with the rest of the time spent navigating or managing their accounts (panel C). Only 7% (100/1432) of clinicians spent less than half their time online reading.

The first and last clicks of most clinicians (773/1432, 54%) were ≥48 weeks apart (panel D). In other words, more than half of the sample used UpToDate for, essentially, the full year. Only 5% (72/1432) of clinicians used UpToDate for 4 weeks or less over the year. Overall, lags were brief; 73% (1045/1432) of clinicians logged on to UpToDate within 1 week of receiving the activation email, and 88% (1260/1432) logged on within 4 weeks of receiving the email. Another 31% (444/1432) of clinicians dropped out; their use lapsed for 6 or more weeks before the close of the study period (data not shown).

On average, clinicians were active on UpToDate for 30% of the days in the period between their first and last sessions in the year. For example, clinicians whose first and last sessions were 90 days apart logged on to UpToDate on 30 of those days on average. Overall, 3% (43/1432) of clinicians were active nearly every day during their period of use (panel E). On the days that clinicians logged in to UpToDate, 80% (1146/1432) spent 3 or more minutes online on average, 9% (129/1432) spent more than 10 minutes online, and the highest user spent 26.6 minutes online. The median number of minutes per active day of use was 4.4 (panel F).

### Defining Segments

First, we attempted to capture the intensity of use of UpToDate among enrolled clinicians. We compared the total number of sessions with the total time spent online and found them to be very highly correlated (correlation coefficient: 0.97), suggesting that either measure could stand in for the other in the definition of behavioral segments. We decided to focus on time spent online because we believe that it is more intuitively representative of clinician activity than the number of sessions. To capture the intensity of this time online, we focused further on time spent online per active day (panel F). To simplify this distribution, we wanted to create a binary classification of clinicians based on “light” and “heavy” rates of use. The median rate of use per active day was 4.4 minutes, which was close to 5 minutes—a round and intuitive number that would split the sample more or less evenly.

**Figure 4:**
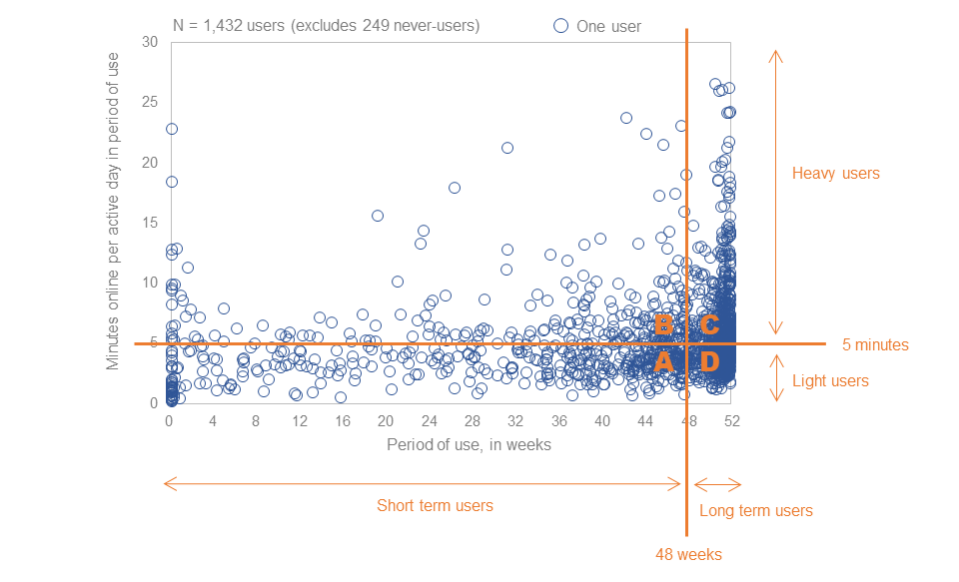
Segment definitions.

Second, we wanted behavioral segments to reflect the period of use of UpToDate according to the calendar. Approximately half of all clinicians 54% (773/1432) used UpToDate for 48 weeks or more—roughly the full year (panel D). From this information, we constructed another binary variable designating “long-term” users, whose period of use was 48 weeks or more, and all other “short-term” users. This classification is easily interpretable as clinicians who used UpToDate for roughly the full subscription year versus those who used it for less than the full subscription year (Figure 4).

We defined 4 segments based on these two binary variables (light or heavy use and short-term or long-term use) and a final segment containing the never-users who had no online behaviors, as follows:

- Segment A (lower left quadrant): short-term, light users (25%, 420/1681)
- Segment B (upper left quadrant): short-term, heavy users (15%, 252/1861)
- Segment C (upper right quadrant): long-term, heavy users (24%, 403/1681)
- Segment D (lower right quadrant): long-term, light users (22%, 370/1681)
- Segment E: never-users (15%, 352/1681)

### Descriptive Statistics of Segments

**Figure 5:**
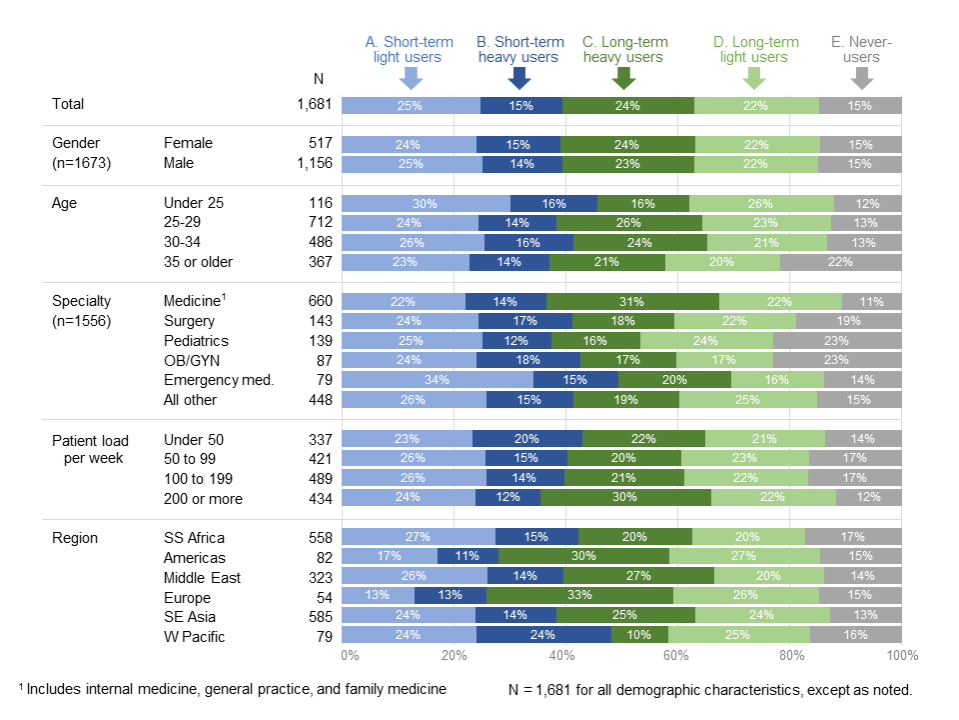
Distribution of segments by demographic and practice characteristics. OB/GYN: obstetrician-gynecologist; SE: southeast; SS; Sub-Saharan; W: west.

Among the full sample, 25% (420/1681) of applicants were short-term, light users; 15% (252/1681) of applicants were short-term, heavy users; 24% (403/1681) of applicants were long-term, heavy users; 22% (370/1681) of applicants were long-term, light users; and the remaining 15% (252/1681) of applicants never logged on to the tool at all. Segment distribution was very similar among men and women; however, the distribution varied somewhat by age group, specialty, and patient load per week (Figure 5). For instance, applicants aged 35 years or older were more likely to be never-users, while those under 25 years of age were more likely to be short-term, light users. Those specializing in medicine (including internal medicine, general practice, and family medicine) were the most likely to be long-term, heavy users, whereas those specializing in emergency medicine were the most likely to be short-term, light users. Applicants with high patient loads (200 or more per week) were more likely to be long-term, heavy users and less likely to be never-users compared to those with lower patient loads.

Segment distribution varied much more strongly by region (Figure 5). Applicants from the Americas and Europe were more likely to be long-term, heavy users compared to, for example, those in other regions.

### Baseline Survey Data

**Figure 6:**
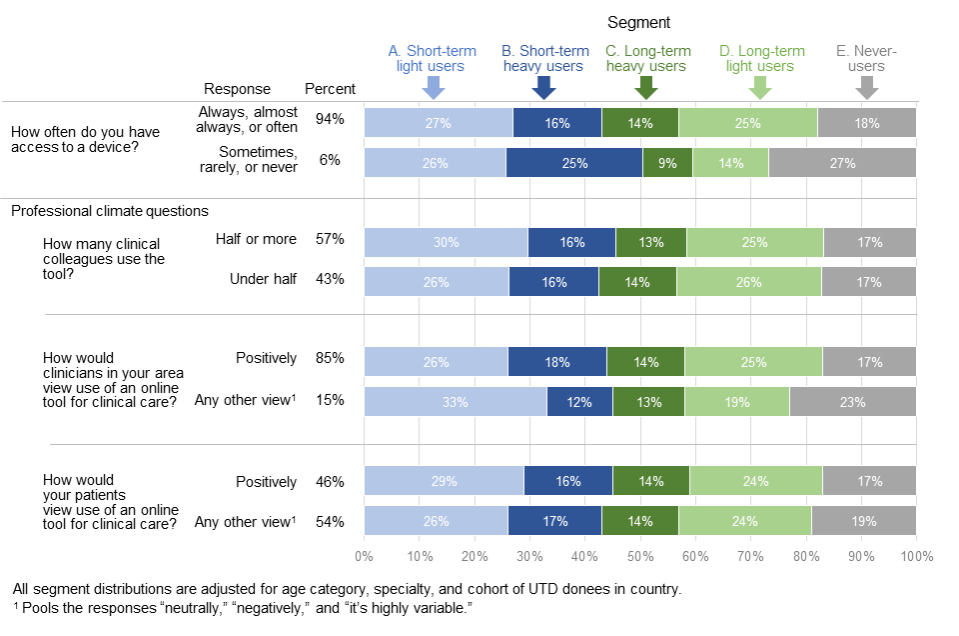
Relationship between baseline facilitators and online activity over the full year. UTD: UpToDate.

At baseline, 94% (1580/1681) of applicants reported that they always, almost always, or often had access to a device on which they could use the EBCR. The remaining 6% (101/1681) had less frequent access to a device, and these applicants were more likely than others to be never-users (454/1681, 27% vs 303/1681, 18%) or short-term users (857/1681, 51% vs 723/1681, 43%; Figure 6).

As for professional climate, 57% (958/1681) of clinicians reported that half or more of their clinical colleagues used an online clinical resource at baseline, but user segment distribution did not differ greatly for those reporting less use of an online clinical resource among colleagues. Most respondents (1429/1681, 85%) reported that clinicians in their area would view the use of an online tool positively, and compared to those who reported other views, these respondents were less likely to be never-users (286/1681, 17% vs 387/1681, 23%) and more likely to be long-term users (655/1681, 39% vs 370/1681, 22%). Nearly half of the users (773/1681, 46%) reported that they thought that patients would positively view the use of an online tool during clinical care. The segment distribution of this group was similar to the complementary group of clinicians who thought that patients would view the use of a tool neutrally, negatively, or variably (Figure 6).

## Discussion

### Principal Findings

#### Overview of Segments

In this study, we developed a method for behaviorally segmenting users of an established digital health tool based on their clickstream data. From a sample of 1681 clinicians in 75 countries, we defined 5 segments of users based on their online behavior. This involved performing extensive technical work to preprocess the raw clickstream data and identify patterns of use, resulting in the synthesis of 3 million clicks into a handful of segments. The value of the segments, in turn, is to support implementation efforts to increase the utility of EBCRs in low-resource settings. Understanding more about the meaning and behavior of each segment can help drive uptake.

User segments are often based solely on demographic characteristics, such as age, gender, or geography, and while we did find some variation in segments based on these traits, they are not precise correlates of online behavior. Clinicians aged over 35 years, for example, are more likely than younger clinicians to never log on, yet 88% (1479/1681) of them did log on at some point, and 21% (353/1681) were long-term, heavy users. In this way, these behavioral segments add distinct information about clinician users beyond demographics.

These segments also reveal the impact of barriers to access that clinicians may face even before they receive the donated subscription. We find, for example, that clinicians who have less frequent access to a device are more likely to never log on and less likely to be long-term users, suggesting that if we work toward improving access to devices, we may remove a powerful barrier to use. We also asked about barriers regarding professional climate in 3 ways. With regard to these three aspects, the perceived attitudes of fellow clinicians in the area were most strongly related to the following segment: those who believed that other clinicians viewed the use of ECBRs positively were more likely to log on at all and be long-term users compared to those who believed otherwise. This relationship was weaker based on perceptions of patient attitudes, suggesting that interventions for increasing use should focus on trying to change clinician attitudes rather than patient attitudes.

In this way, these segments can richly inform efforts to implement EBCRs in low-resource settings. Each segment itself suggests different needs.

#### Segment A: Short-Term, Light Users

Segment A clinicians logged in but did not use UpToDate for the full year and used it for less than 5 minutes per day when they did. They may have had challenges with slow internet, managing the search and navigation tools, implementing results in English, or paying for data. They may benefit from having a local collaborator demonstrate the value of the tool, receiving targeted communication materials from the Better Evidence program, or other early interventions to enable them to overcome these barriers. Discovering more about the barriers that this segment faced will allow us to design interventions for bypassing such hurdles.

#### Segment B: Short-Term, Heavy Users

Segment B clinicians did not use the tool for the full year, but on days when they logged on, they used it for 5 minutes or more. They may have been involved in a research project, may have found the tool useful, or may have been excited to improve their practice. The short-term nature of their use may be related to a loss of access to a device, the loss of a password or login, the cost of data related to use, or a change in position or job. Studying both the barriers and facilitators of use in this segment will provide additional insights into generating excitement for the tool’s use and into bypassing contextual hurdles.

#### Segment C: Long-Term, Heavy Users

Segment C clinicians used the tool for the full year and for longer than 5 minutes on the days that they logged on. Heavy use may be related to patient load, disease burden, or other job responsibilities. Segment C clinicians could be strong advocates for EBCRs; they could explain the value of the tool to colleagues or mentor segment A clinicians. Studying segment C clinicians will reveal the facilitators to EBCR usage in various contexts.

#### Segment D: Long-Term, Light Users

Segment D clinicians used the tool over the full year, with fewer than 5 minutes online per active day. They may be specialists who do not see a wide array of conditions and therefore have fewer topics to review, or they may be skilled navigators who find the answers to their questions quickly. They also may only be using certain features of the tool. Segment D clinicians might benefit from learning about additional features that they have not yet explored.

#### Segment E: Never-Users

Segment E clinicians never logged in to their subscription at all. They may change email addresses between the time of application and the time of award, they may not recognize or see the subscription email, or they may lose interest between the time of application and the time of receipt. The option to update one’s email address while pending application review, clearer explanations of how the subscription award notice will arrive, or follow-up emails with nonusers may increase participation, reduce dropout, and improve the value of the program. Survey responses may also further inform how we can better serve segment E clinicians and their patients.

Using these behavioral segments to implement program changes will require further research. In forthcoming analyses, we will join these clinicians’ clickstream data with additional longitudinal survey data on barriers and facilitators. In the future, in-depth qualitative interviews with clinicians from each segment may yield useful information for ensuring uptake.

### Limitations

This study faces some limitations. First, these clickstream data arose from a convenience sample of Better Evidence donation recipients who enrolled over a 9-week period. This sample may not be representative of all Better Evidence recipients or all digital health tool users working in resource-limited settings or with vulnerable populations. Second, we followed these users for a 12-month period, but multiple years of data may reveal more about user behavior. Third, many sessions did not have an “ending event,” requiring an imputation approach. In the aggregate, the distribution of imputed values reasonably approximated the length of the sessions, but as with any imputation method, they may not have been exact at the individual level.

### Conclusions

Digital health tools promise to improve health care delivery across the globe, including in traditionally underserved areas, in isolated settings, and for clinicians serving vulnerable populations. However, without appropriate and active implementation, it is likely that resource gaps with regard to digital health will widen rather than narrow. Ensuring that we implement digital health tools successfully and measure their impact accurately requires new methods for analyzing the big data generated by such tools. Other fields, such as e-commerce, have developed methods for using these kinds of data; global health must do the same to ensure that digital health tools equip clinicians with essential evidence for providing the best possible care to patients and populations. We believe that the user segments we have defined can be used broadly to better implement digital health tools (eg, improve the onboarding process and increase retention), thereby expanding their impact and reach.

## Appendix

Multimedia Appendix 1 Events and activities.

Multimedia Appendix 2 Survey questions.

Multimedia Appendix 3 Countries represented in the sample of users by region.

## Abbreviations

EBCR: evidence-based clinical resource
REDCap: Research Electronic Data Capture
