# Supplementary material for: Segmenting Clinicians’ Usage Patterns of a Digital Health Tool in Resource-Limited Settings: Clickstream Data Analysis and Survey Study
Source: JMIR Form Res. 2022 May 9;6(5):e30320. doi: 10.2196/30320 (PMC9127647; doi:10.2196/30320)
Supplement: Multimedia Appendix 1 [file formative_v6i5e30320_app1.docx]

Title: Segmenting clinicians’ usage patterns of a digital health tool in limited resource settings: methodology and initial results

**S1 Online Supplement: Events and activities**

| Activity | Event type |
| --- | --- |
| Navigating | "FindInTopic" |
|  | "Search/Lucene" |
|  | "Search/Unidex" |
|  | "SearchFacetChange" |
|  | "TocView" |
|  | "TopicView/outline" |
|  | "TopicView/rollover" |
|  | "ImageView/graphicssearch" |
|  | "ToggleSearchResultsViewClick" |
| Reading | "AbstractView" |
|  | "ImageView" |
|  | "ImageView/powerpoint" |
|  | "LocalApp/Get/Abstract" |
|  | "LocalApp/Get/Graphic" |
|  | "LocalApp/Get/Topic" |
|  | "TopicSectionView" |
|  | "TopicView/full" |
|  | "TopicView/text" |
|  | "TopicContributorView" |
|  | "TopicPeerReviewView" |
|  | "DrugInteractionAddDrug" |
|  | "DrugInteractionAnalyzeClick" |
|  | "DrugInteractionLink" |
|  | "DrugInteractionMonographClick" |
|  | "DrugInteractionClearListClick" |
| Account management | "LanguageSelectionClickEvent" |
|  | "MyAccount" |
|  | "MyUpToDateClearHistory" |
|  | "MyUpToDateDeleteBookmark" |
|  | "MyUpToDateShowWidget" |
|  | "SLAClickEvent" |
|  | "WhatsNewChangeEvent" |
| Continuing current activity | "TopicView/print" |
|  | "ImageView/print" |
|  | "ShowMoreResultsClick" |
|  | "MyUpToDateAddBookmark" |
|  | "ContentShare" |
| Ending current activity | “TopicViewEnd" |
|  | "ExternalLinkClick" |
|  | "AbstractPubMedTextClick" |
|  | "DrugPubMedTextClick" |
|  | "SLACloseButtonClickEvent" |
