# Supplementary material for: Segmenting Clinicians’ Usage Patterns of a Digital Health Tool in Resource-Limited Settings: Clickstream Data Analysis and Survey Study
Source: JMIR Form Res. 2022 May 9;6(5):e30320. doi: 10.2196/30320 (PMC9127647; doi:10.2196/30320)
Supplement: Multimedia Appendix 2 [file formative_v6i5e30320_app2.docx]

Title: Segmenting clinicians’ usage patterns of a digital health tool in limited resource settings: methodology and initial results

**S2 Online Supplement: Survey questions**

**From the application for donation**

These are questions from the application to the UpToDate donation program, excluding unique identifiers such as name or email address. These questions are asked as a routine part of the donation program, independent of this research study.

* Items with asterisks were required to complete.

| **Item** | | **Response options** | | | |
| --- | --- | --- | --- | --- | --- |
| What is your age? * | | Number | | | |
| How many years of clinical experience do you have? | | Number | | | |
| Country where you work with the organization * | | Drop-down list | | | |
| Your organization is: *  Check all that apply. | | A government agency  A university, college, or other education  A non-governmental organization (NGO)  A public hospital  A mission hospital  A physician solo practice  A group/family practice  Other | | | |
| Where does funding/revenue for your organization's services come from? *  Check all that apply. | | Government  International donors (PEPFAR, USAID, DFID, Global Fund, etc.)  Patients' insurance  Patients' payments and fees  Private philanthropy  Other | | | |
| Is your organization in a rural or urban setting? *  Mark only one oval. | | Mostly urban  Mostly rural  All rural  All urban  50/50 | | | |
| What is your role/profession? *  Mark only one oval. | | Physician  Surgeon  Resident  Attending  Medical Doctor  Medical Registrar  House Officer  Medical Officer  Medical Intern | | Medical Specialist  Physician Assistant  Nurse  Nurse Practitioner  Pharmacist  Corporate  Medical Librarian  Medical Student  Other | |
| What is your medical specialty?  Mark only one oval. | Allergy and immunology  Anesthesiology  Cardiology  Dermatology  Emergency medicine  Endocrinology  Family medicine  Gastroenterology  General practice  Geriatrics  Hematology  Hospital medicine | | Infectious disease  Internal medicine  Nephrology  Neurology  OB/GYN  Oncology  Ophthalmology  Orthopedic surgery  Otorhinolaryngology  Palliative care  Pathology | | Pediatrics  Psychiatry  Pulmonary  Radiology  Rheumatology  Sleep medicine  Sports medicine  Surgery  Urology  Women's health  Other: |
| Tell us why you need a donated subscription: | | | | | |
| In a short paragraph, please tell us more about your work: *  Please describe the mission of your organization, why and when you got involved, and what you work on. | | | | | |
| In a short paragraph, please tell us why you should receive a donated UpToDate subscription and its potential impact on the community you serve. * | | | | | |

**From the baseline survey**

These are questions from the baseline survey administered to applicants after the UpToDate donation questions and informed consent. These were asked only during the enrollment window for the study, and are no longer collected from applicants for donations. The four questions presented here are the ones used in this analysis; the full survey is longer. Data from other questions will be reported in future publications of results.

How often do you have access to a smartphone, tablet or computer while providing clinical care?

a. Never

b. Rarely

c. Sometimes

d. Often

e. Almost always

f. Always

Approximately how many of the clinical care providers that you work with use UpToDate? (Select one)

a. 100%

b. 75%

c. 50%

d. 25%

e. 0%

f. I don’t know

g. N/A (I don’t work with other clinical providers.)

|  | Negatively | Neutrally | Positively | It’s highly variable | I don’t know |
| --- | --- | --- | --- | --- | --- |
| How do you think clinicians in your area would view the use of an online tool like UpToDate for clinical care? | 1 | 2 | 3 | 4 | 5 |
| How do you think your patients would view the use of an online tool like UpToDate during clinical care? | 1 | 2 | 3 | 4 | 5 |
