# Supplementary material for: Segmenting Clinicians’ Usage Patterns of a Digital Health Tool in Resource-Limited Settings: Clickstream Data Analysis and Survey Study
Source: JMIR Form Res. 2022 May 9;6(5):e30320. doi: 10.2196/30320 (PMC9127647; doi:10.2196/30320)
Supplement: Multimedia Appendix 3 [file formative_v6i5e30320_app3.docx]

Title: Segmenting clinicians’ usage patterns of a digital health tool in limited resource settings: methodology and initial results

**S3 Online Supplement: Countries represented in the sample of users, by region**

| Sub-Saharan Africa  World Health Organization classification: African region (AFR) | | |
| --- | --- | --- |
|  | Angola | Mozambique |
|  | Botswana | Namibia |
|  | Burundi | Nigeria |
|  | Cameroon | Rwanda |
|  | Chad | Sierra Leone |
|  | Democratic Republic of the Congo | South Africa |
|  | Ethiopia | Swaziland |
|  | Ghana | Tanzania |
|  | Kenya | Togo |
|  | Liberia | Uganda |
|  | Madagascar | Zambia |
|  | Malawi | Zimbabwe |
|  | Mauritius |  |
| Americas  World Health Organization classification: Region of the Americas (AMR) | | |
|  | Argentina | Honduras |
|  | Bolivia | Jamaica |
|  | Brazil | Mexico |
|  | El Salvador | Nicaragua |
|  | Guatemala | Peru |
|  | Guyana | Venezuela |
|  | Haiti |  |
| Middle East  World Health Organization classification: Eastern Mediterranean Region (EMR) | | |
|  | Afghanistan | Pakistan |
|  | Iraq | Palestine |
|  | Jordan | Somalia |
|  | Kuwait | South Sudan |
|  | Libya | Sudan |
|  | Morocco | Syria |
|  | Oman | Yemen |
| Europe  World Health Organization classification: European Region (EUR) | | |
|  | Armenia | Romania |
|  | Belarus | Russia |
|  | Kosovo | Turkey |
|  | Kyrgyzstan | Ukraine |
| Southeast Asia  World Health Organization classification: South-East Asian Region (SEAR) | | |
|  | Bangladesh | Myanmar (Burma) |
|  | Bhutan | Nepal |
|  | India | Sri Lanka |
|  | Indonesia | Timor-Leste |
|  | Maldives | Cambodia |
| West Pacific  World Health Organization classification: Western Pacific Region (WPR) | | |
|  | China | Papua New Guinea |
|  | Fiji | Philippines |
|  | Laos | Vietnam |
